# The Influence of Radiographic Phenotype and Smoking Status on Peripheral Blood Biomarker Patterns in Chronic Obstructive Pulmonary Disease

**DOI:** 10.1371/journal.pone.0006865

**Published:** 2009-08-31

**Authors:** Jessica M. Bon, Joseph K. Leader, Joel L. Weissfeld, Harvey O. Coxson, Bin Zheng, Robert A. Branch, Venkateswarlu Kondragunta, Janet S. Lee, Yingze Zhang, Augustine M. K. Choi, Anna E. Lokshin, Naftali Kaminski, David Gur, Frank C. Sciurba

**Affiliations:** 1 Division of Pulmonary, Allergy and Critical Care Medicine, Department of Medicine, University of Pittsburgh, Pittsburgh, Pennsylvania, United States of America; 2 Department of Radiology, University of Pittsburgh, Pittsburgh, Pennsylvania, United States of America; 3 Department of Epidemiology, University of Pittsburgh, Pittsburgh, Pennsylvania, United States of America; 4 James Hogg iCAPTURE Center, Vancouver General Hospital, Vancouver, British Columbia, Canada; 5 Department of Clinical Pharmacology, University of Pittsburgh, Pittsburgh, Pennsylvania, United States of America; 6 Division of Pulmonary and Critical Care, Department of Medicine, Brigham and Women's Hospital, Harvard Medical School, Boston, Massachusetts, United States of America; 7 University of Pittsburgh Cancer Institute, University of Pittsburgh, Pittsburgh, Pennsylvania, United States of America; Emory University, United States of America

## Abstract

**Background:**

Chronic obstructive pulmonary disease (COPD) is characterized by both airway remodeling and parenchymal destruction. The identification of unique biomarker patterns associated with airway dominant versus parenchymal dominant patterns would support the existence of unique phenotypes representing independent biologic processes. A cross-sectional study was performed to examine the association of serum biomarkers with radiographic airway and parenchymal phenotypes of COPD.

**Methodology/Principal Findings:**

Serum from 234 subjects enrolled in a CT screening cohort was analyzed for 33 cytokines and growth factors using a multiplex protein array. The association of serum markers with forced expiratory volume in one second percent predicted (FEV1%) and quantitative CT measurements of airway thickening and emphysema was assessed with and without stratification for current smoking status. Significant associations were found with several serum inflammatory proteins and measurements of FEV1%, airway thickening, and parenchymal emphysema independent of smoking status. The association of select analytes with airway thickening and emphysema was independent of FEV1%. Furthermore, the relationship between other inflammatory markers and measurements of physiologic obstruction or airway thickening was dependent on current smoking status.

**Conclusions/Significance:**

Airway and parenchymal phenotypes of COPD are associated with unique systemic serum biomarker profiles. Serum biomarker patterns may provide a more precise classification of the COPD syndrome, provide insights into disease pathogenesis and identify targets for novel patient-specific biological therapies.

## Introduction

While airflow obstruction is the hallmark of chronic obstructive pulmonary disease (COPD), the distinct processes of parenchymal destruction and small airway fibrosis can induce similar impairments in expiratory flow. Varying contributions of airway remodeling and emphysema can be found in any individual, leading to significant heterogeneity in disease expression [1], [2], [3], [4]. The precise classification of these disease phenotypes is paramount to the elucidation of pathogenic mechanisms and the development of innovative, effective therapies for COPD. In fact, future research efforts will be immobilized without a more thorough understanding of the molecular pathogenesis driving phenotype expression and a more refined schema for characterizing disease.

Although the presence of systemic inflammation in COPD is widely accepted [4], [5], [6], [7], the inter-individual variation in the patterns of the inflammatory response is not emphasized in the existing literature. The diverse presentation of disease resulting from a single environmental exposure, cigarette smoke, suggests the presence of distinct bio-molecular and cellular pathways leading to a divergence of anatomic phenotypes. However, the variation of peripheral inflammatory mediators and the association with pulmonary histopathologic phenotypes remains a largely uninvestigated research area. Whereas the recent evolution of quantitative computed tomography (CT) technology has provided a non-invasive method of estimating the contribution of airway thickening versus parenchymal emphysema within an individual patient [2], [8], [9], [10], [11], we postulated that characterization of the inflammatory profile within radiographic sub-types of COPD will provide a basis for the refinement of disease classification, offer insights into variations in disease pathogenesis and identify novel therapeutic targets and surrogates associated with clinically meaningful outcomes. Therefore, we conducted a cross-sectional study examining both the association of unique serum inflammatory profiles with quantitative CT derived airway and parenchymal phenotypes of COPD and the variation of these associations with smoking status.

## Methods

The following is an abridged version of the methods section. Please refer to Methods S1 for detailed methodology.

### Ethics Statement

The study protocol was approved by the University of Pittsburgh Institutional Review Board. Participating subjects provided written informed consent for research use of their CT scans and blood samples.

### Subject Selection

Two hundred and thirty-four participants were selected from the Pittsburgh Lung Screening Study (PLuSS) cohort. Participants were current or former smokers ages 50–79 and were selected to represent the spectrum of visual radiographic emphysema and airflow obstruction (Table S1, Table S2, Table S3). Subjects with a restrictive spirometric pattern, history of lung cancer, or suspicion of lung cancer at screening were excluded from the study. All subjects were ambulatory and self-referred from a mass-mailing recruitment effort.

### Pulmonary Function Testing

Spirometry was performed on all subjects upon entry into the PLuSS cohort. Testing was performed using standard methodology [12], [13] and reference equations [14].

### Quantitative CT Analysis

The subjects underwent low-dose CT examinations performed on either a LightSpeed Plus 4-detector (n = 110) or LightSpeed Ultra 8-detector (n = 124) (GE Healthcare). The CT examinations were acquired using a helical technique at 120 or 140 kVp with a mean tube current-time product of 28.9 (+7.9) mAs. Images were reconstructed contiguously at 2.5 mm section thickness with a 2.5 mm interval using a GE Healthcare high-spatial frequency kernel with a range of pixel dimensions from 0.54 to 0.98 mm.

The apical bronchus of the right upper lobe was manually selected from the CT images and analyzed in cross-section. Wall area as a percentage of total airway area (WA%), which has been associated with lung function [15], [16], was computed using a partial membership algorithm developed at the University of Pittsburgh [17], [18] and used as a measure of bronchial thickening. The lung depicted in CT images was segmented [19] and the extent of emphysema was assessed using the density-mask technique [9]. Parenchymal voxels with computed attenuation values less than −950 Hounsfield Units (HU) were defined to be associated with emphysema. The volume of lung associated with emphysema was represented as the fraction voxels less than the −950 HU threshold of the total computed lung volume (F-950).

### Serum biomarker measurements

Stored serum samples were analyzed for thirty-three serum chemokines and growth factors using a bead-based cytometric immunoassay system (Luminex, Austin, TX). A detailed description of the methods of the multiplex assay performed at the Core facility has been described previously by others [20]. Standard curves were generated for each cytokine in concordance with the manufacturer's instructions and the concentrations of unknown samples were calculated using a 5 parametric curve-fitting program with logistic regression (Bio-Rad Laboratories, Hercules, CA).

### Statistical Analysis

Continuous data were summarized as either mean ± standard deviation or median and quartiles and categorical data were expressed as percentages. The association between the forced expiratory volume in the first second percent predicted (FEV1%) and WA% and F-950 was analyzed using univariate linear regression analysis. The contribution of WA% and F-950 to FEV% was then determined using multiple regression analysis. Finally, the relationship between WA% and F-950 was assessed with the Pearson's correlation coefficient.

Serum biomarkers with concentrations above or below the detection threshold of the assay were respectively assigned the highest or lowest extrapolated value for that given marker (Table S4). Because the data was not normally distributed, biomarker levels were log-transformed and the association between the log-transformed values and FEV1%, WA% and F-950 for the entire cohort was assessed using univariate linear regression analysis and multiple regression analysis after adjusting for smoking status. Linear regression analysis stratified for smoking status was then performed to evaluate the association of serum inflammatory markers and FEV1%, WA% and F-950 separately in current and former smokers. All statistical procedures were performed using SAS version 9.1.

## Results

### Subject demographics

The selected cohort consisted of 149 current and 85 former smokers (Table 1). The subjects' FEV1% ranged from 15 to 134% with a mean of 68.2% (±27.1%). The mean subject age was 61.3 years with a range of 50 to 78 years.

**Table 1:** Subject Characteristics by GOLD Classification.

Characteristics	GOLD Category				
	At Risk	1	2	3	4
	(n = 56)	(n = 38)	(n = 58)	(n = 64)	(n = 18)
**Age**	59.1±7.2	62.1±8.0	61.7±7.2	62.5±7.1	60.6±6.5
**Sex**					
Male	25 (45%)	22 (58%)	29 (50%)	33 (52%)	7 (39%)
Female	31 (55%)	16 (42%)	29 (50%)	31 (48%)	11 (61%)
**Current Smoker**	30 (54%)	27 (71%)	43 (74%)	41 (64%)	8 (44%)
**FEV1 (%)**	98.4±13.0	92.1±8.4	67.2±8.0	40.9±5.9	23.8±4.8
**FEV1/FVC (%)**	76.9±4.5	63.5±4.3	59.6±7.5	44.2±7.3	32.7±6.5

### Quantitative CT analysis

**Figure 1:**
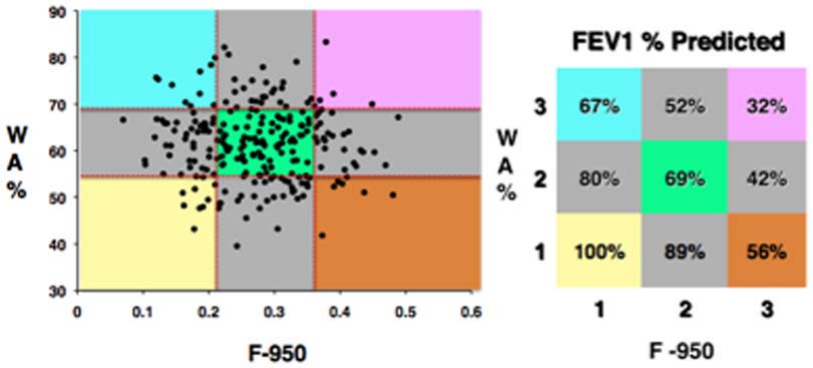
Scatter plot of the fraction of CT voxels with attenuation values less than −950 Hounsfield Units (F-950) plotted on the X-axis and the right upper apical lobe bronchus wall area as a percentage of total airway area (WA%) plotted on the Y-axis. Tertiles of F-950 and WA% are demarcated by the dotted vertical and horizontal lines within the scatter plot with the mean FEV1 percent predicted (FEV1%) values represented in the corresponding color-coded grid. No association exists between F-950 and WA% (r = −0.055, p = 0.40) and the severity of parenchymal emphysema and airway disease cannot be predicted based on FEV1 alone.

Both WA% and F-950 were correlated with FEV1% in univariate analysis (r = −0.39, p = <0.0001; r = −0.43, p = <0.0001) and each CT parameter independently contributed to FEV1 decline in multivariate analysis (r = 0.6). Although there was a strong association of airway thickening and parenchymal emphysema CT phenotypes with FEV1%, there was no association of the CT phenotypes to each other (r = −0.055, p = 0.40) suggesting that each parameter represents an independent, unique phenotype (Fig. 1).

### Association between serum inflammatory markers and FEV1%

Four of the 33 markers were modestly associated with FEV1% (p = <0.05) before and after adjustment for current smoking status (Table 2). Eotaxin, matrix metalloproteinase (MMP)-1, and MMP-7 were inversely associated with FEV1%. Epidermal growth factor receptor (EGFR) showed a direct association with FEV1% and thus decreased with increasing GOLD classification.

**Table 2:** Association Between Log Transformed Serum Markers and FEV1 Percent Predicted (N = 234).

Analyte	Co-Efficient (log pg/ml / % predicted)	P-Value
Eotaxin	−17.09	0.04
MMP-1	−8.55	0.02
MMP-7	−18.19	0.02
EGFR	42.16	0.05

### Association between serum inflammatory markers and WA%

Seven of the 33 biomarkers were associated with WA%. Interleukin (IL) -6, IL-13, IL-2 receptor, Interferon gamma (IFN-γ), and c-reactive protein (CRP) were directly associated with WA% while regulated on activation normal T cell expressed and secreted (RANTES) was inversely associated with this CT parameter (Table 3, Table S5). Notably none of these markers demonstrated independent association with FEV1. On the other hand, consistent with its direct relationship with FEV1%, EGFR decreased as WA% increased. All relationships held with adjustment for current smoking status. Notably, none of the above markers, except IL-6, demonstrated an association with F-950 despite their association with airway wall thickness.

**Table 3:** Association Between Log Transformed Serum Markers and Percent Wall Area (N = 234).

Analyte	Co-Efficient (log pg/ml / WA%)	P-Value
IL-2R	3.17	0.003
IL-6	1.58	0.01
IL-13	4.68	0.009
IFN-γ	3.03	0.01
EGFR	−12.65	0.04
RANTES	−1.76	0.05
CRP	2.36	0.03

### Association between serum inflammatory markers and F-950

Three of the 33 inflammatory markers, IL-6, MMP-7 and tumor necrosis factor alpha (TNF-α), were associated with quantitative emphysema (Table 4, Table S5). While IL-6 and MMP-7 were directly associated with F-950, TNF-α was inversely related to emphysema severity. Again, adjustment for smoking status had little effect on the association of biomarkers and emphysema. Of note, MMP-7 showed consistent associations across both FEV1% and F-950, demonstrating an inverse relationship with FEV1% and a direct relationship with F-950 but not WA%.

**Table 4:** Association Between Log Transformed Serum Markers and Percent Emphysema (N = 234).

Analyte	Co-Efficient (log pg/ml / F-950)	P-Value
IL-6	0.016	0.02
TNF-α	−0.011	0.04
MMP-7	0.047	0.04

### The interaction of serum inflammatory markers and smoking

Stratified analysis by smoking status revealed a relationship between specific biomarkers and FEV1% (Table 5) that differs based on smoking status. The association of eotaxin, EGFR, MMP-1, and MMP-7 with obstruction was significant in former, but not current, smokers.

**Table 5:** Association Between Log Transformed Serum Markers and FEV1 Percent Predicted Stratified by Current Smoking Status.

Analyte	Co-Efficient (log pg/ml / % pred)	P-Value	Co-Efficient (log pg/ml / % pred)	P-Value
	Former n = 85		Current n = 149	
EGFR	68.07	0.04	19.28	0.49
Eotaxin	−32.78	0.02	−6.97	0.51
FAS-L	−34.53	0.02	4.27	0.64
MMP-1	−16.91	0.01	−3.95	0.39
MMP-7	−29.58	0.009	−7.30	0.48

**Figure 2:**
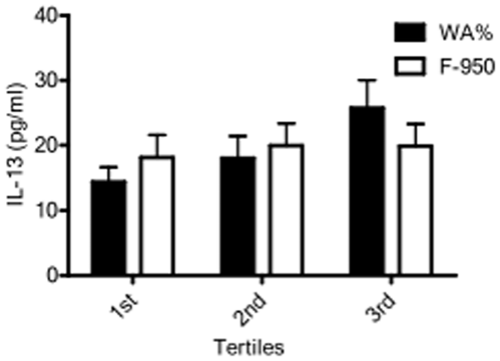
Bar graph representing mean (standard error of the mean) interleukin 13 (IL-13) serum levels between tertiles of wall area percentage (WA%) and emphysema (F-950). IL-13 serum levels increase with increasing tertiles of WA% with the highest levels occurring in those subjects with the most airway thickening (p = 0.038). IL-13 serum levels do not significantly vary between tertiles of F-950.

**Figure 3:**
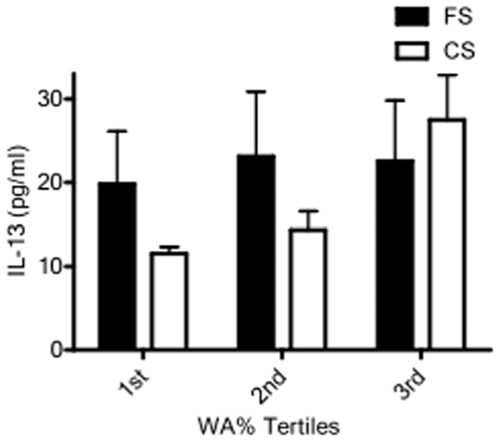
Bar graph representing mean (standard error of the mean) interleukin 13 (IL-13) serum levels between tertiles of wall area percentage (WA%) stratified by smoking status. IL-13 serum levels increase significantly with increasing WA% tertile only in current smokers (p = 0.003). Former smokers do not exhibit a significant change in IL-13 levels with degree of airway thickening (FS = former smokers, CS = current smokers).

**Figure 4:**
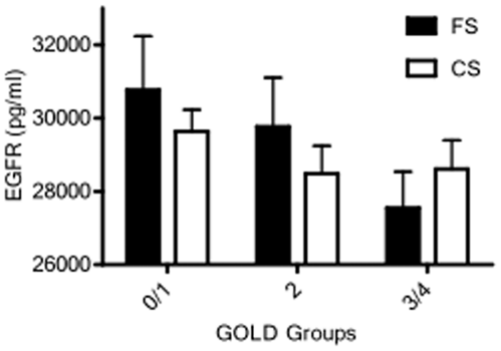
Bar graph representing mean (standard error of the mean) soluble epidermal growth factor receptor (EGFR) serum levels between GOLD groups stratified by smoking status. EGFR serum levels decrease with increasing GOLD group in former smokers (p = 0.16) but do not significantly vary with severity of obstruction in current smokers (FS = former smoker, CS = current smoker).

Likewise, the relationship between serum biomarkers and WA% in current versus former smokers demonstrated associations dependent on current smoking status (Table 6). While IL-13 concentrations were directly associated with WA% (Table 3) and increased with increasing WA% tertiles (Fig. 2), these relationships were only observed in current smokers (Table 6; Fig. 3). Similarly, a significant association of IL-6, IFN-γ, and CRP with WA% was present only in current smokers (Table 6). This is in contrast to serum EGFR concentrations, which were directly associated with FEV1 (Table 5) and showed a trend toward decreasing with increasing GOLD classification in former, but not current, smokers (Fig. 4). MMP-2, MMP-7 and TNF-α receptor two (TNF-RII) were also significantly associated with WA% in ex-smokers alone. Notably, subjects' smoking status did not affect the associations between serum inflammatory markers and quantitative emphysema.

**Table 6:** Association Between Log Transformed Serum Markers and Wall Area Stratified by Current Smoking Status.

Analyte	Co-Efficient (log pg/ml / WA%)	P-Value	Co-Efficient (log pg/ml / WA%)	P-Value
	Former n = 85		Current n = 149	
IL-2R	3.81	0.01	2.69	0.06
IL-6	0.23	0.84	2.16	0.006
IL-13	2.51	0.33	6.46	0.008
IFN-γ	1.85	0.28	3.97	0.01
CRP	0.47	0.79	3.47	0.01
EGFR	−19.86	0.02	−6.24	0.47
TNF-RII	10.14	0.03	0.78	0.82
MMP-2	18.33	0.05	−7.94	0.41
MMP-7	8.01	0.006	−3.07	0.34

## Discussion

Recently, the paradigm has shifted away from viewing COPD as a single disease entity to viewing it as a heterogeneous syndrome with variable contributions of peripheral airway fibrosis and emphysema. Our study illustrates this heterogeneity in a cohort of high-risk smokers and demonstrates systemic inflammatory patterns distinctive to individual anatomic phenotypes. Although the presence of a generalized systemic inflammatory response in individuals with both stable and progressive COPD has been well established [4], [5], [6], [7], only recently have studies explored the relationship between inflammatory biomarkers and distinct disease phenotypes. Elevation of select biomarkers have been implicated in COPD patients with increased resting energy expenditures [21] and skeletal muscle loss [22]. Blood markers or genetic polymorphisms have been shown to vary independently with functional capacity, severity of dyspnea, diffusing capacity, and BODE score [23], [24], [25]. To our knowledge, no study has explored the variation of serum inflammatory markers in association with CT indices of emphysema and airway disease.

The severity of airway remodeling and parenchymal emphysema can be characterized with quantitative CT analysis. Histological-radiological correlate studies have demonstrated a relationship between the extent of low attenuation areas depicted on CT images and histologic emphysema [8], [9]. Quantitative CT analysis of low generation airways have also been shown to correlate with disease of the small airways [26], the dominant site of airway resistance in COPD [27]. Similar to others [2], [16], [28], we found a significant contribution of quantitative CT estimates of airway remodeling and parenchymal emphysema to FEV1 in smokers with a wide spectrum of airflow obstruction, supporting the coexistence of two unique CT phenotypes that correlate with disease severity and vary remarkably between individuals.

Our findings of distinct inflammatory patterns associated with airway thickening and parenchymal emphysema supports the existence of unique biological processes contributing to the syndrome of COPD. Of the markers demonstrating a significant association with airway thickening, only EGFR demonstrated an association with FEV1 and only IL-6 was simultaneously associated with parenchymal emphysema. Because our study focused on the measurement of serum biomarkers, we can not know whether the patterns of peripheral inflammation reflect a “spill-over” of the local inflammatory milieu within the lungs or represent a synchronous, systemic molecular diatheses associated with either airway or parenchymal disease. The significant but modest associations imply that a complex interplay of chemokines and growth factors, rather than one or two inflammatory mediators, is associated with the development of individual phenotypes.

In a complex disease where pathogenic processes progress after cessation of cigarette smoking [29], we have also demonstrated inflammatory marker associations with airway remodeling and obstruction severity that varied according to current smoking status. Both animal and human studies have shown increased inflammatory proteins in the presence of cigarette smoke [30], [31], [32] and prior groups have found smoking-dependent differential associations between inflammatory proteins and markers of disease severity [33]. Relevant to our finding of a direct association of IFN-γ and IL-13 with WA% in current, but not former, smokers, others have also demonstrated interactions between smoking and both IFN-γ [34] and IL-13 [35] polymorphisms in association with lung function. We have further defined specific associations with CT measures of airway thickening independent of airflow obstruction. This differential relationship suggests that, while inflammation may persist following smoking cessation, the specific biological processes may differ from that in current smokers. Alternatively, underlying processes associated with either innate [36] or adaptive immune responses to colonization or autoimmunity[37], [38] may be dominated by the inflammatory effects of tobacco smoke. Such associations emphasize the complexity of the inflammatory process underlying the pathophysiology of COPD.

Although the multiple simultaneous measurements enabled by the high-throughput nature of Luminex technology increases the probability of type I error, we did not correct for multiple comparisons in our analysis. A formal adjustment would minimize the number of false positive findings but would also increase the probability of missing clinically meaningful associations. In this exploratory study, we did not want to overlook possible relationships and instead focused on those markers that demonstrated both biological plausibility and consistency in their associations with CT parameters. For instance, we found that IL-13, a cytokine associated with lung [39] and airway inflammation and fibrosis [40] in animal models and lung function in human studies [25], [35], [41], was directly related to WA% in our study cohort. Although IL-13 and FEV1% were not inversely associated in this study, we did find a significant indirect relationship in a separate cohort of individuals with COPD [25]. EGFR, a soluble growth factor receptor which has been shown to be decreased in individuals with breast carcinoma [42], non-small cell lung carcinoma, and head and neck carcinoma [43] and to be lower with increased melanoma tumor burden [44], appeared to have a protective effect in our study cohort – levels increased with increasing FEV1% and decreasing WA%. The biological plausibility of IL-13 and the consistency across EGFR relationships suggests clinically meaningful associations that are not merely a result of increased type-I error. Although the number of plausible associations we have observed are statistically unlikely to have occurred randomly, we fully acknowledge that any individual markers identified must be further validated in other ongoing patient cohorts [45]. Likewise, as CT technologies evolve enabling analysis of multiple, higher generation airways, further validation studies will be necessary to either confirm these molecular associations or to determine whether analysis of the right upper lobe apical bronchus versus smaller airways provides independent, meaningful information.

In conclusion, our study illustrates the heterogeneity of the COPD syndrome exemplified by independent variability of airway remodeling and parenchymal destruction and demonstrates an association between distinct quantitative CT phenotypes and serum inflammatory biomarker patterns. Many of these associations varied according to current cigarette use, suggesting a complex interplay of inflammation and environment leading to the variable expression of airway disease and obstruction. The traditional definition of chronic airflow obstruction fails to recognize the diversity of biologic processes represented by varying patterns of disease expression, which most likely will vary in response to molecular therapeutics. The challenge is to develop tools that precisely classify individuals based on their unique pathophysiologic phenotypes. Whereas quantitative CT indices of emphysema and airway remodeling have been shown to correlate with physiology and histology, the identification of inflammatory markers that segregate with anatomic phenotypes further validates and facilitates more robust disease sub-classification and provides further insight into molecular-cellular mechanisms, potential therapeutic targets and easily measured surrogates of disease activity.

## Supporting Information

Methods S1 (0.04 MB DOC)

Table S1 Distribution of eligible subjects by GOLD classification and semi-quantitative emphysema score (0 = none, 1 = trace, 2 = mild, 3 = moderate, 4 = severe) N = 3297
(0.03 MB DOC)

Table S2 Distribution of subjects randomly selected from each of the 9 strata formed by cross-classification according to GOLD and emphysema score N = 260
(0.03 MB DOC)

Table S3 Distribution of final subjects selected for analysis by GOLD and emphysema score N = 234
(0.03 MB DOC)

Table S4 Number of out-of-range (OOR) values and values assigned for 33 markers
(0.05 MB DOC)

Table S5 Regression co-efficients for associations between log-transformed serum analyte levels and WA and F-950
(0.05 MB DOC)
