# Supplementary material for: The Influence of Radiographic Phenotype and Smoking Status on Peripheral Blood Biomarker Patterns in Chronic Obstructive Pulmonary Disease
Source: PLoS One. 2009 Aug 31;4(8):e6865. doi: 10.1371/journal.pone.0006865 (PMC2730536; doi:10.1371/journal.pone.0006865)
Supplement: Methods S1 — (0.04 MB DOC) [file pone.0006865.s001.doc]

**S1 - METHODS**

The following is an unabridged version of the methods section.

***Subject Selection***

Two-hundred and thirty-four participants were selected from the Pittsburgh Lung Screening Study (PLuSS) cohort. Participants were current or former smokers ages 50-79 and were selected to represent the spectrum of visual radiographic emphysema and airflow obstruction. All subjects were ambulatory and self-referred from a mass-mailing recruitment effort and had no past personal history of lung cancer. The entire PLuSS population included 3724 enrolled subjects. Participants were excluded based on the following criteria: missing pulmonary function test (PFT) data (N=16), a lung cancer diagnosis at screening or during follow-up (N=45), a collection of fewer than two buffy coat aliquots (N=78) or a collection of fewer than four aliquots (N=177), a history of smoking less than one-half pack cigarettes per day (N=103) or of smoking fewer than 25 years (N=126), missing visual semi-quantitative measurement of centrilobular emphysema on low-dose CT scan (N=423), or restrictive PFT results (FEV1/FVC greater than 70% and an FEV1% of less than 80%; N=451). Based on these criteria, 1069 subjects with one or more exclusion criteria were excluded, leaving 2655 eligible subjects.

Eligible subjects were stratified according to GOLD classification and semi-quantitative emphysema score (0=none, 1=trace, 2=mild, 3=moderate, 4=severe) (Table S1). Two-hundred sixty subjects, frequency matched by gender, were randomly selected from each of the nine strata formed by cross-classification according to GOLD and Emphysema Score (Table S2). Intentional over sampling from strata representing extreme situations (e.g. moderate or severe emphysema with no more than mild airflow obstruction (GOLD 1) or no emphysema with moderate or severe airflow obstruction (GOLD 3 or 4)) was utilized to enhance selection of airway-dominant and emphysema-dominant phenotypes. Twenty-six subjects were then excluded due to missing Luminex data or obesity, leaving 234 subjects available for final analysis (Table S3).

The study protocol was approved by the University of Pittsburgh Institutional Review Board. Participating subjects had provided informed consent for research use of their CT scans and blood samples as part of the initial PLuSS consent process.

***Pulmonary Function Testing***

Spirometry was performed on all subjects upon entry into the PLuSS cohort. Testing was performed using standard methodology [1, 2] and standard reference equations [3].

***Quantitative CT Analysis***

The 234 subjects underwent low-dose CT examinations that encompassed the entire lung and were performed on either a LightSpeed Plus 4-detector (n=110) or LightSpeed Ultra 8-detector (n=124) CT scanners (GE Healthcare). The CT examinations were acquired using a helical technique at 120 or 140 kVp with a mean tube current-time product of 28.9 (+ 7.9) mAs. The CT images were reconstructed contiguously at 2.5 mm section thickness with a 2.5 mm interval using a GE Healthcare high-spatial frequency kernel with a range of pixel dimensions from 0.54 to 0.98 mm.

The apical bronchus of the right upper lobe was manually selected from the CT images and analyzed in cross-section. Wall area as a percentage of total airway area (WA%), which has been associated with lung function [4, 5], was computed using a partial membership algorithm developed at the University of Pittsburgh [6, 7] and used as a measure of bronchial thickening. The lung depicted in CT images was segmented [8] and the extent of emphysema was assessed using the density-mask technique [9]. Parenchymal voxels with computed attenuation values less than -950 Hounsfield Units were defined to be associated with emphysema. The volume of lung associated with emphysema was represented as the fraction voxels less than the -950 HU threshold of the total computed lung volume (F-950).

***Serum biomarker measurements***

Serum samples were obtained from subjects at entry into the PLuSS cohort. Thirty-three serum chemokines and growth factors were measured using a bead-based cytometric immunoassay system (Luminex, Austin, TX, USA) (Table S4). A detailed description of the methods of the multiplex assay performed at the Core facility has been described previously by others [10]. Five batches of plates were used to assay the 33 inflammatory markers. The first batch consisted of 23 cytokines in multiplex format (Biosource Invitrogen, Camarillo, CA). The second batch consisted of EGFR, FAS, and FASL analytes in multiplex format (University of Pittsburgh Luminex Core Facility, Pittsburgh, PA). The third batch consisted of metalloproteinases in multiplex format (R&D Systems, Minneapolis, MN). The fourth and fifth batch measured CRP concentrations and MPO concentrations respectively (LINCO Research, St. Charles, MO). Standard curves were generated for each cytokine in concordance with the manufacturer’s instructions and the concentrations of unknown samples were calculated using a 5 parametric curve fitting program with logistic regression (Bio-Rad Laboratories, Hercules, CA). Standard curves for VEGF and FAS ligand were consistently poor, therefore these markers were excluded from the final analysis.

***Statistical Analysis***

Continuous data were summarized as either mean ± SD or median and quartiles and categorical data were expressed as percentages**.** The association between FEV1% and WA% and F-950 was first analyzed using simple linear regression analysis with FEV1% as the dependent variable. The contribution of WA% and F-950 to FEV% was then determined using multiple regression analysis. Finally, the relationship between WA% and F-950 was assessed with the use of Pearson’s correlation coefficient.

Serum biomarkers with concentrations above or below the detection threshold of the assay were respectively assigned the highest or lowest extrapolated value for that given marker. Because the data was not normally distributed, serum biomarker levels were log transformed and the association between the log transformed values and FEV1%, WA% and F-950 for the entire cohort was assessed initially using simple linear regression analysis and multiple regression analysis after adjusting for smoking status. Linear regression analysis stratified for smoking status was then performed to evaluate the association of serum inflammatory markers and FEV1%, WA% and F-950 separately in current and former smokers. All statistical procedures were performed using SAS version 9.1.

**REFERENCES**

1. (1991) Lung function testing: selection of reference values and interpretative strategies. American Thoracic Society. Am Rev Respir Dis 144(5):1202-18.

2. (1995) Standardization of spirometry, 1994 update. American Thoracic Society. Am J Respir Crit Care Med 152(3):1107-36.

3. Hankinson JL, Odencrantz JR, Fedan KB (1999) Spirometric Reference Values from a Sample of the General U.S. Population. Am. J. Respir. Crit. Care Med. 159(1):179-187.

4. Nakano Y, Muro S, Sakai H, Hirai T, Chin K, et. al. (2000) Computed Tomographic Measurements of Airway Dimensions and Emphysema in Smokers . Correlation with Lung Function. Am. J. Respir. Crit. Care Med. 162(3):1102-1108.

5. McNitt-Gray MF, Goldin JG, Johnson TD, Tashkin DP, Aberle DR (1997) Development and testing of image-processing methods for the quantitative assessment of airway hyperresponsiveness from high-resolution CT images. J Comput Assist Tomogr 21(6):939-47.

6. Leader JK, Zheng B, Sciurba F, Coxson H, Fuhrman C et. al. (2007) Quantification of airway morphometry: the effect of CT acquisition and reconstruction parameters. Proc SPIE 6511:1R-1 to 1R-7.

7. Zheng, B, Leader J, McMurray J, Park S, Fuhrman C et. al. (2007) Automated detection and quantitative assessment of pulmonary airways depicted on CT images. Med Phys 34(7):2844-2852.

8. Leader, J, Zheng B, Rogers R, Sciurba F, Perez A et. al. (2003) Automated lung segmentation in x-ray computed tomography: development and evaluation of a heuristic threshold-based scheme. Acad Radiol 10(11):1224-1236.

9. Muller, N, Staples C, Miller R, Abboud R (1988) 'Density Mask'. An objective method to quantitate emphysema using computed tomography. Chest 94:782-787.

10. Gorelik, E, Landsittel DP, Marrangoni AM, Modugno F, Velikokhatnaya L et. al. (2005) Multiplexed Immunobead-Based Cytokine Profiling for Early Detection of Ovarian Cancer. Cancer Epidemiol Biomarkers Prev 14(4):981-987.
