# Supplementary material for: The Influence of Radiographic Phenotype and Smoking Status on Peripheral Blood Biomarker Patterns in Chronic Obstructive Pulmonary Disease
Source: PLoS One. 2009 Aug 31;4(8):e6865. doi: 10.1371/journal.pone.0006865 (PMC2730536; doi:10.1371/journal.pone.0006865)
Supplement: Table S1 — Distribution of eligible subjects by GOLD classification and semi-quantitative emphysema score (0 = none, 1 = trace, 2 = mild, 3 = moderate, 4 = severe) N = 3297 (0.03 MB DOC) [file pone.0006865.s002.doc]

|  | **Semi-quantitative** |  |  |
| --- | --- | --- | --- |
|  | **Emphysema Score** |  |  |
|  | **0** | **1/2** | **3/4** |
|  |  |  |  |
| **At risk/GOLD 1** | n=1572 | n=670 | n=87 |
|  |  |  |  |
| **GOLD 2** | n=304 | n=317 | n=131 |
|  |  |  |  |
| **GOLD 3/4** | n=57 | n=73 | n=84 |
|  |  |  |  |
|  |  |  |  |
|  |  |  |  |

**Table S1. Distribution of eligible subjects by GOLD classification and semi-quantitative emphysema score (0=none, 1=trace, 2=mild, 3=moderate, 4=severe) N=3297**
