# Supplementary material for: The Influence of Radiographic Phenotype and Smoking Status on Peripheral Blood Biomarker Patterns in Chronic Obstructive Pulmonary Disease
Source: PLoS One. 2009 Aug 31;4(8):e6865. doi: 10.1371/journal.pone.0006865 (PMC2730536; doi:10.1371/journal.pone.0006865)
Supplement: Table S3 — Distribution of final subjects selected for analysis by GOLD and emphysema score N = 234 (0.03 MB DOC) [file pone.0006865.s004.doc]

|  | **Semi-quantitative** |  |  |
| --- | --- | --- | --- |
|  | **Emphysema Score** |  |  |
|  | **0** | **1/2** | **3/4** |
|  |  |  |  |
| **At risk/GOLD 1** | n=49 | n=8 | n=37 |
|  |  |  |  |
| **GOLD 2** | n=30 | n=9 | n=19 |
|  |  |  |  |
| **GOLD 3/4** | n=28 | n=14 | n=40 |
|  |  |  |  |
|  |  |  |  |
|  |  |  |  |

**Table S3. Distribution of final subjects selected for analysis by GOLD and emphysema score N=234**
