# Supplementary material for: The Influence of Radiographic Phenotype and Smoking Status on Peripheral Blood Biomarker Patterns in Chronic Obstructive Pulmonary Disease
Source: PLoS One. 2009 Aug 31;4(8):e6865. doi: 10.1371/journal.pone.0006865 (PMC2730536; doi:10.1371/journal.pone.0006865)
Supplement: Table S4 — Number of out-of-range (OOR) values and values assigned for 33 markers (0.05 MB DOC) [file pone.0006865.s005.doc]

| **Serum Marker** | **Number OOR** | **Assigned OOR** |
| --- | --- | --- |
|  |  |  |
| IL-1β | 134 | 1.65 |
| IL-2 | 74 | 2.55 |
| IL-4 | 91 | 0.61 |
| IL-6 | 42 | 0.22 |
| IL-8 | 13 | 0.17 |
| IL-10 | 222 | 0.8 |
| IFN-γ | 206 | 2.09 |
| TNF-α | 52 | 0.08 |
| Eotaxin | 0 | n/a |
| MCP-1 | 0 | n/a |
| MIP-1α | 37 | 6.78 |
| MIP-1β | 7 | 0.29 |
| Rantes | 30 | 447105.6 |
| EGF | 33 | 0.64 |
| VEGF | 196 | 2.43 |
| FGF-β | 176 | 8.23 |
| G-CSF | 20 | 115.72 |
| HGF | 0 | n/a |
| IL-13 | 196 | 10.19 |
| TNF-RI | 0 | n/a |
| TNF-RII | 0 | n/a |
| MIG | 103 | 12.36 |
| IL-2R | 2 | 0.32 |
| MMP-1 | 4 | 14.74 |
| MMP-2 | 0 | n/a |
| MMP-7 | 1 | 103.06 |
| MMP-12 | 231 | 12.49 |
| MMP-13 | 200 | 20.94 |
| MPO | 0 | n/a |
| FAS | 0 | n/a |
| FAS-L | 0 | n/a |
| EGFR | 0 | n/a |
| CRP | 0 | n/a |

**Table S4. Number of out-of-range (OOR) values and values assigned for 33 markers**
