# Supplementary material for: The Influence of Radiographic Phenotype and Smoking Status on Peripheral Blood Biomarker Patterns in Chronic Obstructive Pulmonary Disease
Source: PLoS One. 2009 Aug 31;4(8):e6865. doi: 10.1371/journal.pone.0006865 (PMC2730536; doi:10.1371/journal.pone.0006865)
Supplement: Table S5 — Regression co-efficients for associations between log-transformed serum analyte levels and WA and F-950 (0.05 MB DOC) [file pone.0006865.s006.doc]

**Table S5. Regression co-efficients for associations between log-transformed serum analyte levels and WA and F-950**

| **Serum Marker** | **WA** | **F-950** |
| --- | --- | --- |
|  |  |  |
| IL-1β | 0.72 | 0.0014 |
| IL-2 | 0.76 | -0.012 |
| IL-2R | **3.17** | -0.0045 |
| IL-4 | 1.07 | 0.0026 |
| IL-6 | **1.58** | **0.014** |
| IL-8 | -1.11 | -0.0035 |
| IL-10 | 0.77 | -0.0036 |
| IFN-γ | **3.03** | 0.0015 |
| TNF-α | -0.45 | **-0.011** |
| Eotaxin | -0.86 | 0.037 |
| MCP-1 | 0.85 | 0.028 |
| MIP-1α | 0.29 | -0.0069 |
| MIP-1β | 0.60 | 0.0023 |
| Rantes | **-1.76** | 0.0015 |
| EGF | -0.24 | 0.0072 |
| G-CSF | 2.08 | -0.022 |
| HGF | 3.65 | 0.0093 |
| IL-13 | **4.68** | -0.0058 |
| TNF-RI | 3.59 | -0.0076 |
| TNF-RII | 3.87 | 0.0029 |
| MIG | 0.78 | -0.0070 |
| MMP-1 | 1.65 | 0.018 |
| MMP-2 | 4.41 | -0.025 |
| MMP-7 | 2.47 | **0.051** |
| MMP-12 | -9.35 | -0.0058 |
| MMP-13 | 0.016 | -0.031 |
| MPO | -1.04 | 0.013 |
| FAS | -0.73 | 0.0017 |
| FAS-L | 1.86 | -0.0074 |
| EGFR | **-12.65** | -0.050 |
| CRP | **2.36** | -0.0052 |

*Significant associations highlighted

p<0.05
